# Ketamine, as adjuvant analgesics for patients with refractory cancer pain, does affect IL-2/IFN-γ expression of T cells in vitro? A prospective, randomized, double-blind study

**DOI:** 10.1097/MD.0000000000006639

**Published:** 2017-04-21

**Authors:** Naibao Zhou, Zhijian Fu, Hao Li, Kaiguo Wang

**Affiliations:** a Department of Pain Management, Shandong Provincial Hospital Affiliated to Shandong University, Shandong University; b Department of Anesthesiology, Shandong Cancer Hospital Affiliated to Shandong University, Shandong Academy of Medical Sciences, Jinan, P.R. China.

**Keywords:** cancer pain, IFN-γ, IL-2, ketamine, T cells

## Abstract

**Background:**

Ketamine has been used as an analgesic adjuvant with morphine in the treatment of refractory cancer pain recently. But both morphine and ketamine have been reported to produce a number of immunomodulatory effects. The current study was performed to assess whether the concentration of ketamine, as adjuvant analgesics for patient with refractory cancer pain, was related to its effect on T cells interleukin-2 (IL-2)/interferon-γ (IFN-γ) expression in vitro.

**Methods:**

Peripheral blood mononuclear cells (PBMCs) were isolated from venous blood of patients with refractory cancer pain over a Ficoll-Hypaque density gradient. T cells were isolated from by positive selection using anti-CD3 beads. T cells were then treated with vehicle (C group), morphine (200 ng/mL, M group), morphine (200 ng/mL), and different dose of ketamine (100, 200, 1000 ng/mL; MK1, MK5, MK10 group) for 24 hours before stimulation with anti-CD3 and anti-CD28. Then supernatant IL-2 and IFN-γ protein analysis, quantitative reverse transcription polymerase chain reaction (RT-PCR) for IL-2 and IFN-γ were done.

**Results:**

There were no significant difference of supernatant IL-2 and IFN-γ among C group, M group, and MK1 group, but the mRNA of M group and MK1 group were decreased compared with C group (*P* < .05). Compared with C group, both of the supernatant protein and the mRNA of MK5 group and MK10 group were all significantly decreased (*P* < .01). Compared with M group, both of the supernatant protein and the mRNA of MK5 group and MK10 group were all decreased (*P* < .05), while supernatant IL-2 and the mRNA of MK10 group were significantly decreased (*P* < .01).

**Conclusion:**

In conclusion, we confirmed that just as morphine, ketamine dose-dependently suppressed IL-2 and IFN-γ of activated T lymphocyte of patients with refractory cancer pain in vitro, but the inhibitory action of low dose ketamine could be neglected.

## 1 Introduction

Pain is very common for cancer patients, and about 15 million people suffer from cancer pain everyday all over the world.^[1]^ And for patients, refractory cancer pain is difficult to treat and has a great influence on the quality of life. The opioid of first choice for moderate to severe cancer pain is morphine,^[2–5]^ and morphine is the standard “step 3” opioid analgesic against which others are measured. However, morphine for long-term use easily leads to tolerance and hyperaesthesia, for the reason that neuropathic cancer pain is resistant to opioid analgesics and the efficacy of opioid analgesics are controversial.^[6–8]^

The non-competitive N-methyl-D-aspartric acid (NMDA) receptor antagonist ketamine is used as an analgesic adjuvant in the treatment of neuropathic and cancer pain.^[9]^ Recently, the administration of ketamine for pediatric cancer pain has been reported.^[10]^ There are a number of reports of successful use of continuous infusion of ketamine in patients with uncontrolled neuropathic cancer pain.^[11–15]^

Opioids are the most potent analgesics, and a great number of studies have convincingly demonstrated that opioids, in particular morphine are immunosuppressive. As well, ketamine has been reported to have immunomodulatory properties that affect immune cells, including macrophages, T cells,^[16]^ and natural killer cells.^[17]^ But the related reports about immunological effect of ketamine on patients with refractory cancer pain were much less.

Therefore, the current study was performed to assess whether the concentration of ketamine, as adjuvant analgesics for patients with refractory cancer pain, was related to its effect on T cells IL-2/IFN-γ expression in vitro.

## 2 Materials and methods

The study was approved by the ethics committee of Shandong Tumour Hospital (Jinan, China) and conducted according to the principles of the Helsinki Declaration. Before inclusion into the study, informed, written consent was obtained from patients with cancer pain, which last for more than 3 months.

### 2.1 The criteria of inclusion and exclusion

The criteria of inclusion: all cases were finally diagnosed malignant tumor, and were received oral analgetica of 3-step analgesic ladder advocated by the WHO, but received less effect; the predicted life span was more than 2 months.

The criteria of exclusion: cases with tumor concurrent infection, ulceration, and pain; cases being dead in 1 month or the therapeutic regimen being changed; cases with dyscrasia, infection, extensively metastased to distant organ, damaged function of liver, heart, and lung.

### 2.2 Isolation and culture of T cells

Blood was withdrawn after venipuncture. Peripheral blood mononuclear cells (PBMCs) were isolated over a Ficoll-Hypaque (Cedarlane, Canada) density gradient according to the manufacturer's recommendations. T cells were isolated by positive selection using anti-CD3 beads (Miltenyi Biotech, CA) following the manufacturer's instructions, and confirmed by fluorescence-associated cells sorting (85% purity). T cells were adjusted to a final concentration of 1 × 10^5^ cells/mL in 96-well plates. T cells were cultivated at 37 °C and 5% CO_2_ in Roswell Park Memorial Institute (RPMI) 1640 medium (Gibco BRL, Maryland) supplemented with 10% fetal calf serum and antibiotics (100 U/mL penicillin and 100 mg/mL streptomycin; Gibco).

### 2.3 Subgroup and intervention of T cells

T cells were then treated with morphine (Humanwell Pharmaceutical Co., China, 200 ng/mL), morphine (200 ng/mL), and ketamine (100 ng/mL, purchased from Sigma–Aldrich as a pure hydrochloride salt without preservatives), morphine (200 ng/mL) and ketamine (500 ng/mL), morphine (200 ng/mL) and ketamine (1000 ng/mL), which were separately marked as M Group, MK1 Group, MK5 Group, and MK10 Group. The process lasted for 24 hours before stimulation with anti-CD3 and anti-CD28 (R&D, Minnesota, 5 μg/mL). Stimulation with anti-CD3 and anti-CD28 lasted for 24 hours. Optimal activation of T cells requires effective occupancy of both the antigen-specific T cells receptor and a second coreceptor, such as CD28.^[18]^ In this study, we investigated the effect of morphine on T cells after stimulation of both anti-CD3 and anti-CD28. Then T cells accepted corresponding management and cytoimmunity indexes were detected.

### 2.4 Supernatant cytokine protein analysis of IL-2 and IFN-γ

Supernatant IL-2 immunoreactive protein concentrations were measured using IL-2 enzyme-linked immunosorbent assay (ELISA) kits which were purchased from Elabscience, Wuhan, China. This ELISA kit uses sandwich ELISA as the method. Assay procedure followed the manufacturer's instructions.

Supernatant IFN-γ immunoreactive protein concentrations were measured using IFN-γ ELISA kits which were purchased from R&D Systems. The R&D OptEIA test is a solid phase sandwich ELISA. Assay procedure also followed the manufacturer's instructions.

### 2.5 Quantitative real-time RT-PCR

Total RNA of T cells was extracted by cells lysis in guanidinium isothiocyanate, followed by phenol acid extraction. One microgram of total RNA was used for cDNA synthesis with Moloney murine leukemia virus reverse transcriptase, RNase H minus (Promega, Germany) and diluted to 50 μL. Two microliters of cDNA was added to each PCR, and amplification was performed with the oligonucleotide primers specific for human IL-2, IFN-γ (BD). IL-2 specific RT-PCR was performed with 5′-GAATGGAATTAATAATTACAAGAATCCC-3′ and 5′-TGTTTCAGATCCCTTTAGTTCCAG-3′ primers, with a preincubation for 10 minutes at 95 °C and 50 cycles with 5 seconds at 95 °C, 5 seconds at 65 °C, and 10 seconds at 72 °C. Quantitative real-time RT-PCR was done in a total volume of 20 μL using a LightCycler-Fast Start DNA Master SYBR Green I kit (Roche, Switzerland) according to the manufacturer's suggestions. IFN-γ specific RT-PCR was performed with 5′-TCGGTAACTGACTTGAATGTCCA-3′ and 5′-TCCTTTTTCGCTTCCCTGTTTT-3′ with a preincubation for 5 minutes at 95 °C and 30 cycles with 30 seconds at 95 °C, 30 seconds at 60 °C, and 30 seconds at 72 °C. Quantitative real-time RT-PCR was done in a total volume of 5 μL using Revert Aid TM First Strand cDNA Synthesis Kit (Roche, Switzerland) according to the manufacturer's suggestions.

## 3 Results

**Figure 1:**
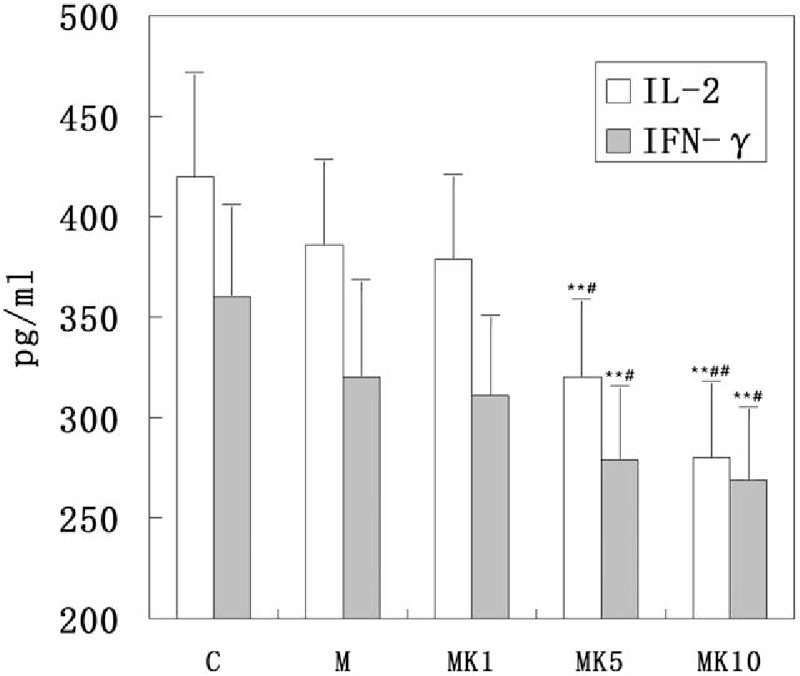
Effects of morphine and ketamine on T cells of patients with refractory cancer pain. PBMCs were isolated over a Ficoll-Hypaque density gradient. T cells were isolated from PBMCs by positive selection using anti-CD3 beads. T cells were then treated with vehicle, morphine (200 ng/mL), and different dose of ketamine (100, 500, 1000 ng/mL) for 24 hours before stimulation with anti-CD3 and anti-CD28. Then supernatant IL-2 and IFN-γ immunoreactive protein concentrations were measured using cytokine-specific enzyme-linked immunosorbent assay kits. ^∗^/^∗∗^, significant at level *P* < .05/.01 compared with the C group; ^#^/^##^, significant at level *P* < .05/.01 compared with the M group. IFN = interferon, IL = interleukin, PBMCs = peripheral blood mononuclear cells.

There were no significant difference of supernatant IL-2 and IFN-γ among C group, M group, and MK1 group. Compared with C group, both of the supernatant protein of MK5 group and MK10 group were all significantly decreased (*P* < .01). Compared with M group, both of the supernatant protein of MK5 group and MK10 group were all decreased (*P* < .05), while supernatant IL-2 of MK10 group were significantly decreased (*P* < .01) (Fig. 1).

**Figure 2:**
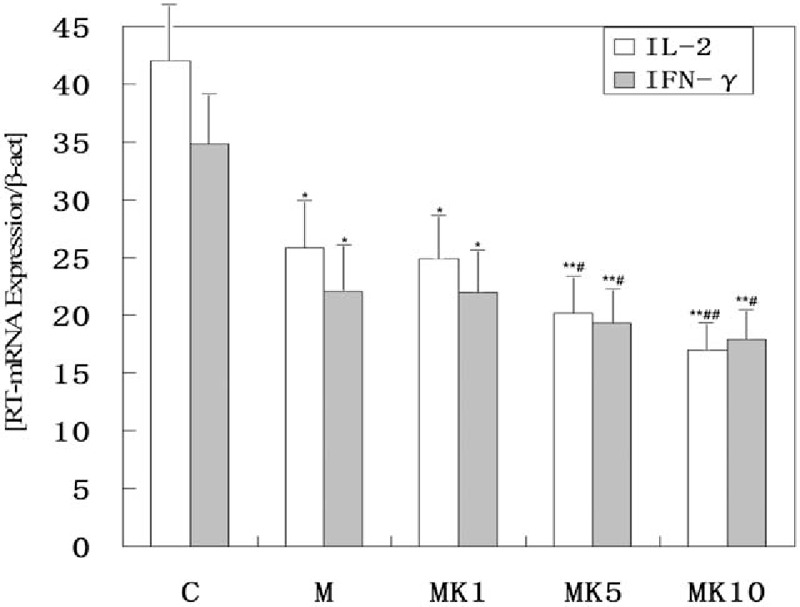
Effects of morphine and ketamine on T cells of patients with refractory cancer pain. PBMCs were isolated over a Ficoll-Hypaque density gradient. T cells were isolated from PBMCs by positive selection using anti-CD3 beads. T cells were then treated with vehicle, morphine (200 ng/mL), and different dose of ketamine (100, 500, 1000 ng/mL) for 24 hours before stimulation with anti-CD3 and anti-CD28. Then total RNA of T cells was extracted by cell lysis in guanidinium isothiocyanate, followed by phenol acid extraction. And amplification was performed with the oligonucleotide primers specific for human IL-2, IFN-γ. Quantitative real-time RT-PCR was done using a LightCycler-Fast Start DNA Master SYBR Green I kit (IL-2) or Revert Aid TM First Strand cDNA Synthesis Kit (IFN-γ). ^∗^/^∗∗^, significant at level *P* < .05/.01 compared with the C group; ^#^/^##^, significant at level *P* < .05/.01 compared with the M group. IFN = interferon, IL = interleukin, PBMCs = peripheral blood mononuclear cells, RT-PCR = reverse transcription polymerase chain reaction.

Compared with C group, IL-2 mRNA, and IFN-γ mRNA were both down-regulated in all groups (*P* < .05), while they were significantly down-regulated in MK5 group and MK10 group (*P* < .01). Compared with M group, IL-2 mRNA and IFN-γ mRNA were both down-regulated in MK5 group and MK10 group (*P* < .05), while IL-2 mRNA were significantly down-regulated in MK10 group (*P* < .01). And in MK1 group, ketamine seemingly has no effect on IL-2 mRNA and IFN-γ mRNA, because the degree of morphine and ketamine decreasing IL-2 mRNA and IFN-γ mRNA wasn’t significantly different from the degree of morphine alone (Fig. 2).

## 4 Discussion

For patients with refractory cancer pain, morphine is the main choice. Its analgesia effect is superior to the other analgesia drug, and its ratio of cost of efficiency is the most optimal analgesic. But morphine for long-term use easily leads to tolerance and hyperaesthesia, and cause severe adverse reaction, such as nausea, vomiting, and hypopnea. In such cases, adjuvant analgesics may be useful, and ketamine is often used for patients with uncontrolled refractory cancer pain.^[11,12,14,15,19]^ In fact, ketamine is often used to treat perioperative pain management^[20–25]^ and procedural pain.^[22–25]^ In addition, the best route of administration of morphine for cancer pain is by infusion.^[10,26–29]^ A variety of doses have been reported: the infusion rates ranging from 0.084 to 0.6 mg/kg/h,^[24]^ compared with subanesthetic doses for pain relief ranging from 0.1 to 1 mg/kg/h.^[10]^ Lately, we linked electronic microdosis infusion pump to epidural catheter (that is patients controlled epidural analgesia, PCEA) to continuously infuse morphine for 15 patients with uncontrolled neuropathic cancer pain, and get good effect. Later, Wang et al^[30]^ linked electronic microdosis infusion pump to veinous catheter (that is patients controlled intravenous analgesia, PCIA) to continuously infuse morphine in complication with ketamine for 42 patients with uncontrolled neuropathic cancer pain, and got good effect, yet. Up to now, we have cured hundreds of this kind of patients. Generally, ketamine alone is not used to treat refractory cancer pain, but it is often used as adjuvant analgesic. Because use of ketamine is short and the scope is limited, we can’t retrieve the standard of ketamine to treat refractory cancer pain.

Opioid drugs can produce tolerance by activating NMDA receptor. A great deal of investigations have indicated that NMDA receptor plays important role in the formation and development of morphine tolerance.^[31,32]^ NMDA receptor is a subtype of excitatory amino acids receptor, and can reinforce excitatory of cells’ response to long time stimulus. It has been found that, NMDA receptor keeps intimate relationship with modulation of nociception, “ribbon-on phenomenon,” primary or secondum hyperalgesia, and neuronic ductility. As noncompetitive NMDA antagonist, ketamine has been certificated to suppress or even turnover morphine tolerance according to animal and clinical experiments.^[24,33–35]^ In addition, ketamine exerts its analgesic effect via attenuation of central sensitization.^[24,34,36]^

The long-term and intensive refractory cancer pain will lead to cacoethic stress reaction and a series of physiopathologic change, including immunologic function. The investigation of Sunagawa et al^[37]^ showed that pain inhibited immunization, and the investigation of Sacerdote et al^[38]^ and Shibasaki et al^[39]^ showed that, too. Therefore, the long-term and intensive refractory cancer pain will lead to cacoethic immune suppression, which will aggravate the state of immune suppression caused by tumor progression and chemoradiotherapy. Accordingly, treating with refractory cancer pain effectively and positively, and relieving immunosuppression have become the pressing duty of we doctors.

As to opioids, there are plenty of studies to prove that, both endogenous opioids, such as β-endorphin, and exogenous opioids, such as morphine, are potent immunomodulators which have inhibitory and stimulatory effects on immune function.^[40–46]^ In vivo, morphine often shows the inhibitory effects, including suppressing a variety of immunocytes: T and NK cells,^[47]^ macrophages, polymorphonuclear leukocytes,^[48,49]^ and lymphocyte,^[50]^ etc. As well, splenic and thymic atrophy have been found in mice receiving morphine.^[51]^ In vitro, morphine triggers T cells apoptosis,^[52]^ and enhances macrophage apoptosis.^[53]^

Ketamine is frequently used intravenous anesthetic, and is the only intravenous anesthetic with analgesic effect. Lots of investigations have declared that ketamine with anaesthetic dose could inhabit function of lymphocyte^[16]^ and NK cells,^[54]^ and lead to metastasis of tumor. Kawasaki et al^[55,56]^ also find that ketamine directly suppressed production of proinflammatory cytokines in the human whole blood. It not only inhabited lipopolysaccharide-induced production of TNF-α, IL-6, and IL-8, but also inhabited TNF-α-induced production of IL-6 and IL-8. Some other study showed that during induction of anesthesia for operation with extracorporeal circulation, 0.25 mg/kg ketamine could obviously inhabit increase of IL-6. Braun et al^[57]^ showed that ketamine at millimolar concentrations induced apoptosis via the mitochondrial pathway. Less toxicity of S(+)-ketamine was observed in neuroblastoma cells, but this difference was minor and, therefore, unlikely to be mediated via the NMDA receptor. Ohta findings^[58]^ suggested that ketamine inhibited the functional maturation of DCs and interferes with DC induction of Th1 immunity in the whole animal. Zeng et al^[59]^ have characterized a variety of effects exerted by ketamine on FLT3-dendritic cells. Ketamine modulated the in vitro and in vivo development of DC subsets, TLR signaling pathways, as well as the downstream T cell response. Gao et al^[60]^ have studied the effect of various concentrations of ketamine (0, 50, 100, 200 μmol/L) on the differenation of human T helper cells in vitro, and found that ketamine dose-dependently decreased TH1 and TH2 cell numbers, TFN-γ and IL-4 level, but increased the ratio of TH1/TH2 and TFN-γ/IL-4 in the presence of phorbol-12-myristate-13-acetate and ionomycin. And recently, Hou et al^[61]^ have proved that with morphine increasing the ratio of CD4^+^/CD8^+^ T cells and Tregs populations, ketamine affected the ratio of CD4^+^/CD8^+^ T cells and Tregs populations of gastric cancer patients in a dose-dependent model in vitro.

In addition, immunosuppression of ketamine is the lowest among the general anesthetic. Subthreshold dose ketamine has little effect on immunocyte and susceptivity of tumor metastasis, and in clinic we use subthreshold dose to relieve effect of ketamine on immune function. Low-dose of ketamine has been confirmed to stimulate activity of immunity cells recently.^[62]^ Beilin et al considered that low-dose of ketamine relieved suppression of immune function due to pain.^[62]^

Cytokines are very important to regulation of immune response, and IL-2 is the most important one because it is involved in T cell regulation, proliferation, and host defense mechanisms. In animal studies, morphine treatment results in a number of immune deficits including significant reduction in IL-2 synthesis. Studies in mouse models have suggested that one of the mechanisms by which opioids cause immunosuppression is the inhibition of IL-2 transcription in activated T lymphocytes.^[63–66]^ As well, produced predominantly in activated T cells,^[67]^ IFN-γ is so important that it modulates all phases of immune processes. And, Wang et al^[68]^ reported that morphine can directly modulate the IFN-γ promoter and alter IFN-γ gene expression and protein synthesis.

In this study, in vitro, without participation of neuroendocrine system, morphine showed direct inhibition of activated T cells. With the effect of morphine, the cytokines, IL-2, and IFN-γ were not significantly decreased, while IL-2 mRNA and IFN-γ mRNA were significantly decreased, from which we can inferred that if the observation was long enough, IL-2 and IFN-γ would significantly decreased. After morphine in combination with low-dose ketamine (100 ng/mL) acting on activated T cells, the trend of quantity change and metergasis of T cell control group, was nearly the same with that of morphine itself, which demonstrated that the inhibition of low-dose ketamine to T cells was tiny.

But with the concentration of ketamine increasing, all of IL-2 and IFN-γ were significantly decreased, regardless of the control group or morphine group compared with. Therefore, we can inferred that ketamine potentially decreased the production of the cytokine of IL-2 and IFN-γ. But the concentrations of ketamine were different from before, which may be related to the design, experiment object, and experiment condition, etc.

## 5 Conclusion

In conclusion, we confirmed that just as morphine, ketamine dose-dependently suppressed IL-2 and IFN-γ of activated T lymphocyte of patients with refractory cancer pain in vitro, but the inhibitory action of low dose ketamine could be neglected. Therefore, in clinic, as adjuvant analgesics for refractory cancer pain, ketamine should be used with the lowest dose, which may be safe and get more profits.
